# The Use of Percutaneous Ultrasound-Guided Radiofrequency Heat Ablation for Treatment of Primary Hyperparathyroidism in Eight Dogs: Outcome and Complications

**DOI:** 10.3390/vetsci5040091

**Published:** 2018-10-18

**Authors:** Rodolfo Oliveira Leal, Laura Frau Pascual, Juan Hernandez

**Affiliations:** 1 Centre Hospitalier Vétérinaire Fregis, 43 Av. Aristide Briand, 94110 Arcueil, France; laurafp90@gmail.com (L.F.P.); juan.hernandez@oniris-nantes.fr (J.H.); 2 CIISA-Centre for Interdisciplinary Research in Animal Health, Faculty of Veterinary Medicine, University of Lisbon, Av. Universidade Técnica, 1300-477 Lisbon, Portugal; 3 Hospital Escolar Veterinário—Faculty of Veterinary Medicine, University of Lisbon, Av. Universidade Técnica, 1300-477 Lisbon, Portugal; 4 Ciutat d’Inca Veterinary Hospital. Av. General Luque, 372, 07300 Inca, Spain; 5 Internal Medecine Unit of Oniris, Nantes-Atlantic College of Veterinary Medicine and Food Sciences, Atlanpole La Chantrerie, CS 40706, 44307 Nantes CEDEX 3, France

**Keywords:** heat-ablation, dogs, hyperparathyroidism, parathyroids, complications, ultrasound, radiofrequency

## Abstract

Percutaneous ultrasound-guided radiofrequency heat-ablation (UG-RHA) is a therapeutic option for dogs with primary hyperparathyroidism (PHPTH) but information about its outcome is still controversial. This retrospective study aimed to evaluate the outcome and complications of UG-RHA in dogs with PHPTH. The medical records of dogs with PHPTH submitted to UG-RHA between June 2012 and September 2015 in a French referral center were retrospectively reviewed. Eight cases were included. No sex predisposition was found. The median age at diagnosis was 12 years. The most common clinical sign was polyuria/polydipsia. All of the dogs were hypercalcaemic prior to UG-RHA, and all showed a parathyroid nodule identified upon cervical ultrasound. UG-RHA was uneventful, allowing a successful resolution of hypercalcemia in all dogs (8/8). Six out of eight dogs did not receive vitamin D supplementation either pre- or post-procedure. From these, three dogs developed biochemical hypocalcemia, but only one required therapy. Other short-term complications included Horner’s syndrome (1/8) and aspiration bronchopneumonia, which led to cardio-respiratory arrest in one large-breed dog (1/8). Long-term complications were scarce, with no recurrence reported in all of the cases that were assessed in follow-up (4/7). This study demonstrates that UG-RHA has few short or long-term complications, and it is a good therapeutic alternative for dogs with PHPTH.

## 1. Introduction

Primary hyperparathyroidism (PHPTH) is an infrequent endocrine disorder in dogs, but it is one of the main common causes of canine hypercalcemia [1,2,3,4,5]. This disease is induced by an overproduction of parathormone (PTH), due to the presence of an adenoma, adenocarcinoma, or an hyperplasia of the parathyroid glands, occurring in 84.9, 5.4, and 12.4% of the cases, respectively [6,7].

The clinical presentation is often vague. The animals can present with polyuria/polydipsia (pu/pd), anorexia, lethargy, weakness, gastrointestinal, or urinary signs [8,9]. In 21% of the cases, hypercalcemia is an incidental finding [8,9]. Depending on the magnitude and duration of hypercalcemia, the clinical signs can progress leading to medical complications such as urolithiasis, which can occur in 24–42% of the cases [4,8,9,10]. The diagnosis of PHPTH is established by measuring a persistent high serum ionized calcium (iCa^2+^) associated with an inappropriate PTH concentration, a normal to low serum phosphate concentration, and by identifying a parathyroid nodule/mass by cervical ultrasonography [1,4].

Several treatments of PHPTH have been described, namely: the surgical removal of the abnormal parathyroid lobe, ultrasound-guided ethanol ablation, or ultrasound-guided radiofrequency heat-ablation (UG-RHA) [1,4]. The success rates are variable, according to different studies. In detail, parathyroidectomy is estimated to be successful in 94% of the treated cases, UG-RHA in 69% to 94%, and chemical/ethanol ablation in 72% to 85% [9,11,12,13,14]. Percutaneous UG-RHA and ethanol ablation are less invasive and usually require a shorter anaesthesia time than the surgical removal of the parathyroid lobe [4,13]. Compared with ethanol, UG-RHA seems to be less traumatic for the surrounding tissues, because the blood flow produces a dispersion of the high temperature, avoiding the direct damage of the vessels [13]. Even if this technique seems particularly safe and less invasive, several disadvantages are recognized. In fact, the nodule size can compromise the technical procedure and the therapeutic success of the UG-RHA treatment. The dogs with small nodules (<2–3 mm) are poor candidates for this procedure, as needle insertion and manipulation can be difficult [4,13,15]. Also, dogs with large parathyroid nodules (>0.35 cm^2^ of cross-sectional area) seem to have a higher rate of recurrence [11]. Consequently, in the cases where small or large nodules are detected, surgical intervention is still recommended. Another disadvantage relies on the inability to have a histological diagnosis of the mass [9,10]. Nonetheless, because parathyroid masses tend to have a benign natural behaviour, their histological classification is not pivotal for the management of PHPTH [16].

Regardless of the treatment modality, hypocalcemia is the most frequent short-term complication, and usually occurs two to three days after the procedure [4,8]. Biochemical hypocalcemia affects around 37% of patients, but only 11% have clinical signs such as facial pruritus, shivering, ataxia, or seizures [9,13,14]. These signs can be controlled by administering vitamin D [1]. Other described complications are temporary dysphagia, hypersalivation, swelling of the surgical area, Horner’s syndrome, and a transient change of voice [9,13,14]. A recurrence of the disease has been described, but it is rare (estimated around 10%) and the overall prognosis is good [1,9].

Although UG-RHA has been increasingly performed in various referral centers, to our knowledge, only four studies have shown the advantages and disadvantages of this technique [11,12,13,14]. Consequently, information regarding their outcomes and complications is still scarce. Therefore, this retrospective study aims to further evaluate the efficacy, and the short-term and long-term complications of percutaneous UG-RHA in dogs with PHPTH.

## 2. Materials and Methods

The medical records of dogs undergoing percutaneous UG-RHA for the treatment of PHPTH, presented to the Centre Hospitalier Vétérinaire (CHV) Fregis (Paris, France), between June 2012 and September 2015, were reviewed. The parameters analyzed included signalment, medical history, physical examination, complete blood count (CBC), biochemistry profile, urine analysis, serum total calcium (tCa^2+^), serum ionized calcium (iCa^2+^), serum phosphate, serum PTH concentration and imaging findings (abdominal ultrasound (US), thoracic radiographs, and cervical ultrasonography). The data concerning the percutaneous UG-RHA, duration of hospitalization, vitamin D supplementation, iCa/tCa follow-up, and short-term (less than one month after the procedure) and long-term complications (more than one month after the procedure) were also assessed.

Percutaneous UG-RHA was performed under general anesthesia (pre-medication with diazepam and butorphanol intravenously (IV), induction with Propofol IV, and intubation and maintenance with volatile isoflurane). The patient was positioned in dorsal recumbency with the neck extended, and the ventral cervical area was clipped and aseptically prepared. The parathyroid mass was identified ultrasonographically, using a sector array transducer from a Philips CX50 ultrasound machine (Philips Ultrasound Andover, MA, USA). An 18-gauge sterile single-use needle was placed into the mass, and radiofrequency energy was applied under ultrasound guidance, by using a monopolar electrocautery. The mean radiofrequency energy of 20 W was initially applied for 90 s, with progressively higher energy frequencies up to 50 W, until all of the tissue became hyperechoic. The needle was redirected several times when necessary, in order to affect the entire mass. All of the procedures were performed by the same board-certified internist (J.H.).

## 3. Results

Eight dogs were included in this study. These were all of the cases diagnosed with PHPTH between June 2012 and September 2015 at CHV Fregis. All of the cases were treated with UG-RHA. The detailed information about each case is presented in Table 1. The median age was 12 years (range 6 to 14 years). There were four males (two neutered) and four females (two neutered). Two dogs were cross-breed; there was one French Bulldog, one German Shepherd, one Labrador, one Jack Russell Terrier, one Great Dane, and one Parson Russell Terrier.

Most of the dogs presented clinical signs, including pu/pd (5/8), decreased appetite (3/8), lethargy (2/8), weakness (2/8), weight loss (2/8), urinary signs (1/8), vomiting (1/8), and shivering (1/8). In three dogs (3/8), no clinical signs were identified, and hypercalcemia was incidentally discovered in a routine blood test. One case was referred for chronic kidney disease and another for gastro-intestinal signs (vomiting and diarrhoea). The other six were referred for an exploration of the hypercalcemia, which had been discovered by the referring veterinarian.

The physical examinations were unremarkable in six out of the eight dogs. One dog presented moderate lethargy and another was moderately dehydrated (6–7%).

No abnormalities were found on the CBC of the included cases. Concerning the biochemical analysis, four out of the eight dogs (4/8) presented a moderate elevation of alkaline phosphatase (ALP: 296, 575, 306, and 110 with a RR of <80 UI/L) and one case had a moderate elevation of alanine-aminotransferase (ALT: 225 UI/L RR: <80 UI/L). Two dogs (2/8; cases 3 and 6 from Table 1) were azotaemic at admission (BUN 1.78 and 2.1 g/L RR: 0.2–0.5 g/L, CREA 2.4 and 1.9 mg/dL, RR 6–16 mg/dL, respectively). Both of the dogs were 12 years-old and only one of them showed hyperphosphatemia (6.2 mg/dL; reference range: 2.5–5 mg/dL). Overall, the serum phosphate concentration was normal to low (median 3.6 mg/dL; reference range: 2.5–5 mg/dL). Concerning the urine analysis, the details were only available for three of the dogs (3/8); urine was inappropriately concentrated in all of these patients (USG of 1.020 in two dogs and 1.017 in one dog); one dog showed calcium oxalate crystalluria and another had a confirmed urinary tract infection. This dog did not undergo percutaneous UG-RHA until the resolution of a urinary tract infection and the negative urine culture. The abdominal USs were unremarkable in six out of the eight dogs; one dog showed a thickened urinary bladder and one had ultrasonographic signs of chronic nephropathy. Information concerning the thoracic radiographies was only available in four dogs, and no significant findings were observed.

All of the dogs had ionized and total hypercalcemia. The median serum tCa^2+^ concentration was 13.0 mg/dL (range 12 to 15.7 mg/L), the median serum iCa^2+^ concentration was 1.87 mmol/L (range 1.47 to 2.60 mmol/L), and the median serum PTH concentration was 526.5 pg/L (range 55 to 3830 pg/mL).

**Figure 1:**
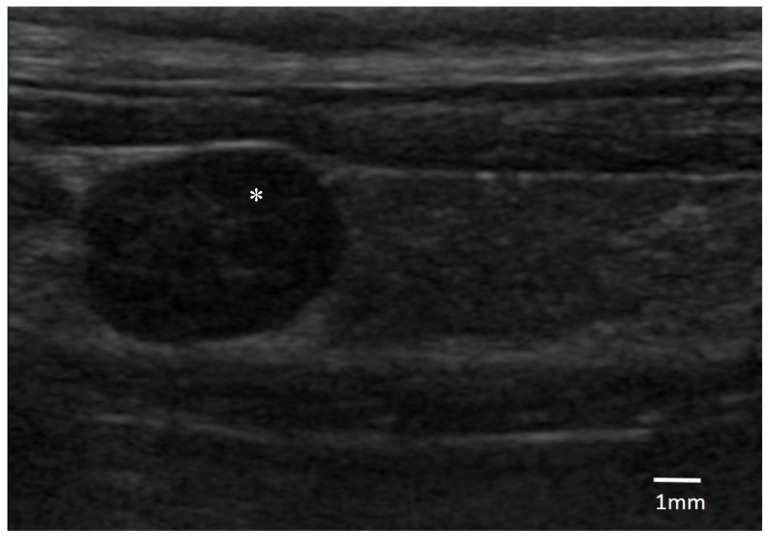
Ultrasonographic image of an oval hypoechogenic parathyroid nodule of 6.5 mm diameter (*), surrounded by thyroid tissue (original, credits to Centre Hospitalier Vétérinaire (CHV) Fregis).

Regarding the cervical US findings, a parathyroid single nodule was identified in all of the dogs (median length 5.3 mm; range 3.5 to 6.5 mm). Five of them were on the right-side, and three on the left. Six nodules were cranially displaced and one was caudal. All of the masses had a similar ultrasonographic aspect being oval, well defined, and hypoechoic in comparison with the surrounding tissues (Figure 1).

**Figure 2:**
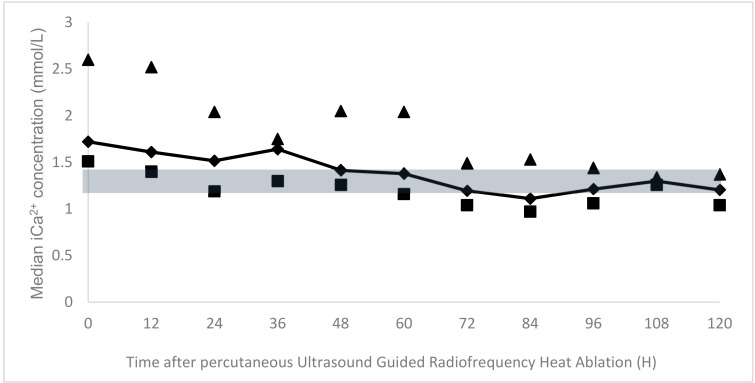
Median serum iCa^2+^ concentrations (dark line) after percutaneous ultrasound-guided radiofrequency heat-ablation (UG-RHA) at different time points, with their respective minimum (squares) and maximum values (triangles). The grey area represents the reference range for iCa^2+^ (1.25–1.45 mmol/L).

The percutanous UG-RHA was uneventful in all of the dogs and allowed for a rapid decrease of serum iCa^2+^ immediately after the procedure (on the same evening). The median serum ionized calcium concentration at discharge was 1.21 mmol/L (range 0.97 to 1.37 mmol/L). Figure 2 illustrates the follow-up of the mean iCa^2+^ at different time-points. The duration of hospitalization ranged from three to nine days (median 4.5 days).

Concerning short-term complications (Table 2), biochemical ionized hypocalcemia was observed in three dogs, in the first three days after the procedure (3/8). Only one of them showed clinical signs, possibly associated with the hypocalcemia (lethargy and generalized weakness), justifying vitamin D supplementation (Calcitriol, 10 ng/kg orally, once daily). From the onset of therapy, the clinical signs progressively reverted. Two dogs were supplemented with vitamin D before the procedure (Calcitriol, 10 ng/kg orally, once daily), and none of them were hypocalcaemic. The Great Dane dog developed lethargy, fever, and dyspnoea 48 h after the procedure. Thoracic radiographies were performed and were compatible with aspiration pneumonia. The coagulation profile revealed an increased prothrombin time (PT) and activated partial thromboplastin time (APTT), and a decreased fibrinogen leading to the suspicion of disseminated intravascular coagulopathy. Despite medical and supportive care, this dog suffered cardiorespiratory arrest and died four days later (six days after the percutaneous UG-RHA). Other short-term complications that were reported were as follows: local inflammation (1/8) and Horner’s syndrome with spontaneous resolution within four weeks (1/8).

Regarding the long-term complications (Table 2), one dog was submitted to euthanasia 40 days after the procedure due to worsening of azotaemia, which was already present before the procedure. Two out of the eight dogs were lost to follow up and four dogs (4/8) had long-term follow-up (variable from 4 to 28 months). The vitamin D therapy was progressively tapered in those dogs taking it and no-one required long-term therapy. Three out of the four cases in which the follow-up was available had a serum iCa^2+^ concentration within the reference range on the last control. In one case, only the tCa^2+^ was monitored, and it was also within the reference range. All of them presented a resolution of the initial clinical signs, and no complications or relapses were seen.

## 4. Discussion

This study retrospectively describes eight cases of PHPTH treated with percutaneous UG-RHA, contributing to an increase in the number of reported cases in the literature. In agreement with previous studies, no sex or breed predispositions were found. All of the patients had an advanced age, reinforcing the idea that PHPTH is often diagnosed in geriatric patients [8]. Although Keeshonds are known to be genetically predisposed for this disease, this breed is not reported in this study, possibly because the breed is rarely seen in France [17].

Similar to what has been previously described in the literature [8,9,10], the most common clinical signs in our study were pu/pd. Although the urine analysis was only detailed in three (3/8) cases, the urinary specific gravity (USG) was inappropriate, reinforcing the reported pu/pd. It is well described that a calcium excess down-regulates the aquaporin-2 water channels, inhibits AVP binding to its receptor site, damaging AVP receptors, and even affecting renal medullary interstitium ion-transport [18]. Consequently, hypercalcemia can induce a decreased USG, as identified in these three cases, as well as in the subsequent pu/pd observed in five of the (5/8) dogs. Urinary tract infection and crystalluria were also identified. These findings are well documented in the dogs with PHPTH and strengthen the need to perform a systematic urine analysis on these patients [4,19].

Concerning the biochemical analyses, 50% of the dogs showed an increase of ALP, which is in agreement with previous studies reporting that it can occur in about 40% of cases [19]. Although the elevation of liver enzymes can be non-specific, it may be secondary to an excess bone resorption and compensatory osteoblastic activity that concurrently occurs in dogs with PHPTH [19]. One case had a moderate non-specific elevation of ALT, which was associated with reactive hepatitis, hepatic ischemia, or concurrent occult liver disease [19]. As this dog was lost to follow up, no further exploration was performed.

Although ionized hypercalcemia was present in all of the animals, the total hypercalcemia did fluctuate, reinforcing that iCa^2+^ is the best parameter to confirm hypercalcemia and monitor the disease [4]. Also, in agreement with previous reports, the serum phosphate concentration was normal to low in these dogs, mainly due to the fact that the parathormone excess induces an increase in the renal excretion of phosphate [2].

Two out of the eight dogs presented azotaemia at presentation, and both were 12-years old. There is some controversy about chronic kidney disease (CKD) in dogs with PHPTH, as it can be either a cause or a consequence, particularly in older dogs. It is well described that when the calcium–phosphate product is greater than 60 mg/dL, soft tissue mineralization can occur and can consequently induce CKD [4]. However, this is unlikely to occur in dogs with PHPTH, as the serum phosphate levels tend to be low. On the other hand, several authors suggest that PHPTH can predispose to kidney injury, and that some dogs with PHPTH can have concurrent CKD [19]. Although the aetiology of azotaemia remains unclear, the fact that, in these two dogs, only a single nodule was identified on cervical US, reinforces the PHPTH suspicion. Consequently, it is believed that chronic hypercalcemia damaged the kidney function, contributing to the progression of CKD.

One of the azotaemic dogs had concurrent hyperphosphatemia. Recognizing that this could eventually compromise the therapeutic success of UG-RHA, the procedure was agreed to by the owners and was performed. Despite the normal ionized calcium after the procedure, the azotaemia and hyperphosphatemia got worse, and the dog was submitted to euthanasia one month after treatment. Further studies are needed to assess whether azotaemia is a contraindication for percutaneous UG-RHA.

A parathyroid isolated hypoechoic nodule was identified in all of the dogs, reinforcing the diagnosis of PHPTH. Although a correlation between the ultrasound and surgical findings can be incongruent in several cases [19], these findings reinforced the value of cervical US in the diagnosis of PHPTH, allowing for a reasonable planning of percutaneous UG-RHA.

UG-RHA was uneventful and allowed for a resolution of ionized hypercalcemia in all of the dogs within the first 48–72 h. Radiofrequency was applied several times, in different needle directions, until the whole tissue became hyperechoic. This was performed in a single procedure and no further interventions were needed. To our knowledge, only four studies reporting percutaneous ultrasound-guided heat ablation have been published. The control of the hypercalcemia was achieved in 69 [15], 72 [13], 90 [14], and 94% [12] of the patients, respectively. The failure of the treatment can be explained by a wrong identification of the parathyroid nodule, inadequate ablation, or the ablation of nodules not secreting PTH [9,11,13]. Also, the importance of operator’s experience has been described [7,9]. In this study, a decrease in the serum iCa^2+^ concentration was observed in all of the dogs, confirming that UG-RHA was successful.

Hypocalcemia was the most frequent short-term complication, in agreement with previous studies [1,8]. Although this is a controversial decision, several authors recommend the supplementation of vitamin D prior to the procedure. One study has reported an inversed correlation between the pre-operative and post-operative total calcium, recommending pre-operative therapy when tCa^2+^ is higher than 3.75 mmol/L (15 mg/dL) [6,7,8]. A recent study also supports this theory, confirming the presence of a moderate correlation between the pre-operative iCa^2+^ value and post-operative hypocalcemia [12]. Conversely, other authors report that the prophylactic administration of vitamin D has no protective value [20] and it should be limited to patients presenting signs of hypocalcemia or a severe decrease in serum calcium concentration after the procedure [21,22]. In the present study, a pre-operative supplementation was performed in only two (2/8) dogs. Not all of the dogs were routinely supplemented, because this clinical decision was not standardized at the hospital, and it was judged individually by the primary internist enrolled in the case before each procedure. Nonetheless, only three patients (3/8) developed transient hypocalcemia, and from these, only one dog required medical management. Therefore, even if it seems prudent to administer vitamin D prior to percutaneous UG-RHA, it did not change the short-term prognosis in this study.

Other complications included Horner’s syndrome and local inflammation. Both had been previously described in former studies [13,14]. Bronchopneumonia (leading to disseminated intravascular coagulopathy and cardiorespiratory arrest) occurred in the Great Dane dog. In fact, it has been described that male large breed-dogs are predisposed to aspiration pneumonia [23]. This complication may have been a consequence of anesthesia and impaired conscious deglutition. Another possible explanation is the eventual involuntary damage of pharyngeal and laryngeal innervation (namely vagus) during UG-RHA, which would have a secondarily predisposition to aspiration pneumonia. Despite the fact that other causes cannot be ruled out (such as pulmonary haemorrhage), the radiographic distribution of the lesions led to the prioritization of aspiration pneumonia. Although it happened in a Great Dane, this complication should alert for the possibility of pharyngeal dysfunction after UG-RHA. Therefore, the dogs submitted to UG-RHA should be closely monitored for the occurrence of respiratory signs, particularly large-breed dogs, which are already prone to aspiration pneumonia.

Regarding long-term complications, even if a recurrence of clinical signs has been previously described [4,7,19], none of these dogs showed clinical or biochemical hypercalcemia. In detail, two dogs were lost to follow up, but four dogs were re-evaluated until the last follow-up (variable from 4 to 28 months), and there were no signs of relapse. These findings reinforce the assessment that percutaneous UG-RHA has an excellent long-term outcome.

This study had some limitations. The main one was the retrospective nature and the difficulty to standardize the data when some medical records were not complete (namely urine analysis and thorax radiographs). Some cases were managed by more than one clinician, which also limited the standardization of the procedures, such as pre-treatment with vitamin D supplementation. Furthermore, the follow-up of several cases was performed by referring veterinarians, which limited the access to further information.

Although it happened in one large-breed dog, to the authors’ knowledge, this is the first study describing bronchopneumonia as a potential short-term complication in dogs with PHPTH submitted to percutaneous UG-RHA.

Besides expanding the number of reported cases in the literature, this study highlights that UG-RHA has few short or long-term complications, and is a good therapeutic alternative in dogs with PHPTH.

## Figures and Tables

**Table 1:** Clinical signs of dogs with primary hyperparathyroidism (PHPTH) submitted to percutaneous ultrasound-guided radiofrequency heat-ablation (UG-RHA). FE—entire female; FN—female neutered; ME—entire male; MN—male neutered.

Case Number	Breed	Age	Sex	tCa^2+^ mg/dL (9–12)	iCa^2+^ mmol/L (1.25–1.45)	PO4 mg/dL (2.5–5.0)	PTH pg/mL (10–200)	Diameter and Side of the Single Parathyroid Nodule
1	Great Dane	9	FN	12.4	1.47	3.1	69	6 mm (right side)
2	Parson Russel Terrier	12	MN	13.6	1.7	4.1	985	2.5 mm (right side)
3	Cross Breed	12	ME	16.3	2.17	6.2	87	6 mm of diameter (right side)
4	Cross Breed	12	FE	13.2	1.58	2.6	185	3.5 mm of diameter (right side)
5	German Shepherd	7	FN	12.3	1.51	2.5	55	6 mm of diameter (right side)
6	Labrador Retriever	12	FE	12.0	2.6	2.7	868	6.5 mm of diameter (left side)
7	French Bulldog	14	ME	12.7	1.87	4.3	3171	5.3 mm of diameter (left side)
8	Jack Russel Terrier	6	MN	15.7	2.6	6.1	3830	5 mm of diameter (left side)

**Table 2:** Short-term and long-term complications identified in dogs with PHPTH submitted to percutaneous UG-RHA.

Short-Term Complications (<1 Month of Duration)	
Biochemical hypocalcemia	**3/8**
Clinical hypocalcemia	**1/8**
Local inflammation	**1/8**
Horner’s syndrome	**1/8**
Aspiration Pneumonia/cardiorespiratory arrest	**1/8**
**Long-Term Complications (>1 Month of Duration)**	
Severe Azotaemia	**1/8**
